# Prevalence and correlates of COVID-19 vaccine hesitancy among the elderly in Qatar: A cross-sectional study

**DOI:** 10.1097/MD.0000000000029741

**Published:** 2022-06-30

**Authors:** Mustafa Abdul Karim, Shuja M. Reagu, Sami Ouanes, Abdul Waheed Khan, Wesam S. Smidi, Nadeen Al-Baz, Majid Alabdulla

**Affiliations:** a Psychiatry Department, Hamad Medical Corporation, Qatar; b Department of Psychiatry, Weill Cornell Medicine, Qatar; c College of Medicine, Qatar University, Qatar.

**Keywords:** COVID-19, elderly, vaccine

## Abstract

Older individuals are more vulnerable to severe coronavirus disease 2019 and medical complications. Vaccination stands as an efficient and safe vanguard against infection. However, negative attitudes and perceptions pertaining to available vaccines might hinder community inoculation. The aim of this study was to assess vaccine hesitancy and its psychosocial determinants among the elderly in Qatar.

We conducted a cross-sectional study between October 15 and November 15, 2020, using a composite online survey including the Vaccine Attitudes Examination Scale in addition to questions on sociodemographic correlates and the role of healthcare professionals.

The vaccine hesitancy rate was 19.5%. The main reasons for willingness to vaccinate included understanding the nature of disease and role of vaccination, in addition to information provided by physicians. Fears mainly centered around vaccine safety. Vaccine hesitators were more likely to be non-Qatari and having received the influenza vaccine at least once. Gender, marital status, socioeconomic status, educational level, and having completed childhood vaccinations were not associated with vaccine hesitancy.

Efforts should be directed toward raising awareness of vaccine efficacy and safety profiles. Physicians should additionally be educated about their pivotal role in advocating vaccine acceptance. We recommend reassessing vaccine hesitancy and its associated factors following a year of campaigning and vaccine administration to identify and target vulnerable groups.

## 1. Introduction

The coronavirus disease 2019 (COVID-19) caused by severe acute respiratory syndrome coronavirus 2 (SARS-CoV-2) was declared a pandemic in March 2020^[1]^ and has so far culminated in excess mortality^[2]^ and unprecedented economic toll on international healthcare facilities.^[3]^ Despite a relatively lower mortality rate compared to previous viral outbreaks, the high infectivity of COVID-19 poses a serious public health threat with no proven highly effective treatments to date.^[4,5]^ Older adults represent a vulnerable group in the face of COVID-19. The case fatality rate for individuals aged 70 to 80 years was estimated to be around 8%, increasing to about 14% for those aged >80 years.^[6]^ Higher prevalence of medical comorbidities such as cardiovascular disease, diabetes mellitus, and chronic kidney disease predisposes this population to more severe infection and medical complications.^[7]^ Spreading awareness to curtail the spread of the virus and advocating vaccine acceptance is hence imperative, particularly in this age group.

Although only a few are mass-produced, over 200 COVID-19 vaccine candidates are presently under different stages of development. Existing evidence highlights the promising results of circulating vaccines and supports further worldwide distribution as benefits outweigh potential risks and adverse effects.^[8]^ Current obstacles crippling global efforts to raise community inoculation against COVID-19 pertain to misinformation, perception, and negative attitudes toward available vaccines. In the United States (US), Kreps et al^[9]^ found that high degrees of vaccine efficacy increased willingness to receive a COVID-19 vaccine, whereas side effect reporting, emergency authorization, and fast-tracking, along with copay decreased willingness. Rzymski and colleagues showed that the highest level of public trust was attributed to mRNA vaccines compared to vaccines developed through different approaches. Fear was mostly related to allergic reactions and adverse events and more observed among females, individuals with lower-level education, and those not seeking information related to the COVID-19 vaccine.^[10]^

In Qatar, a recent cross-sectional study of a nationally representative sample showed that almost a fifth of respondents were unwilling to take the vaccine and 19.8% were unsure. Citizens and females were more likely to be vaccine hesitators. Concerns centered around vaccine safety and possible adverse effects, and the most cited source to raise confidence in available vaccines was personal research.^[11]^ Subanalysis of responses from pregnant and breastfeeding participants revealed a 25% vaccine hesitancy mostly attributed to safety concerns. Further fears reported by this group included infection risk and vaccine shortage.^[12]^ Among healthcare workers, 1 in 8 were unwilling to take the vaccine because of safety and efficacy-related factors, and higher awareness level of the nature of infection and vaccine role predicted acceptance.^[13]^ In this study, we aimed to evaluate vaccine hesitancy and its psychosocial determinants among the elderly population in Qatar.

## 2. Materials and methods

We conducted a national cross-sectional study in Qatar between October 15 and November 15, 2020. We used a composite survey assessing vaccine hesitancy including the Vaccine Attitudes Examination Scale (VAX)^[14]^ in addition to sociodemographic correlates and items pertaining to risks of the new vaccine and role of healthcare professionals. The Arabic translation of the survey was previously validated.^[11]^ The study was approved by the Medical Research Council at Hamad Medical Corporation (MRC- 01-20-930).

### 2.1 Participants

Inclusion criteria comprised all adults aged 65 years or older in the State of Qatar. All nationalities and genders were included. Participants aged <65 years at the time of the study were excluded.

### 2.2 Sample size calculation

We used the Raosoft sample size calculator.^[15]^ In Saudi Arabia, vaccine hesitancy rate was 20.76% (1.89% refused to receive the vaccine, and 20.76% were unsure).^[16]^ With 5% margin of error, 95% confidence level, and estimated population size of 30,000 for individuals aged ≥65 years in the State of Qatar,^[17]^ the estimated sample size was 251.

### 2.3 Statistical analysis

Data were analyzed using IBM SPSS for Windows, version 26.0 (International Business Machines Corporation, Armonk, New York).^[18]^ For categorical variables, we calculated absolute and relative frequencies. For continuous variables (ie, VAX scores), we calculated the mean and the standard deviation. Whenever the variable did not follow the normal distribution, median and interquartile range were calculated. To compare VAX scores among categorical groups, we used the *t* test for independent samples. We constructed a multiple linear regression model with the VAX score as a dependent variable and with the following independent variables: gender, nationality (Qatari vs non-Qatari), marital status, higher education, medical history, psychiatric history, completed childhood vaccination, and history of influenza vaccination. The adjusted *R* square, the unstandardized B coefficient with its 95% confidence interval, the partial correlation coefficient (*R*), and the *P* value were calculated. For all tests, the significance level was set at α = 0.05, and all tests were 2-tailed.

## 3. Results

Our sample consisted of 325 subjects aged ≥65 years. The majority were males, non-Qatari Arabs, married, with university-level education, and retired (Table 1). Most participants completed their childhood vaccinations, and 36.9% received the annual influenza vaccine in the past 3 years. 64.8% of participants reported medical comorbidities, including diabetes mellitus (40.5%), hypertension (48.2%), and dyslipidemia (15.3%). The majority did not report a personal or family history of COVID-19 infection (Table 2). Fears mainly centered around getting infected or having a family member infected with COVID-19 and the unavailability of vaccine (Table 3). In response to the question, “Will you take the COVID-19 vaccination when it becomes available?,” 143 individuals (50.9%) chose “definitely” and 36 individuals (12.8%) reported that they would probably take the vaccine. Forty-seven individuals (16.7%) were not sure, 24 individuals (8.5%) would probably not take the vaccine, and 31 individuals (11.0%) would definitely not take the vaccine. Most individuals believed COVID-19 was a real disease and displayed a willingness to take the COVID-19 vaccine themselves, recommend it to the elderly or family members with chronic conditions, and have their children inoculated when the vaccine becomes available. The main reason for willingness to take the vaccine was understanding the nature of COVID-19 and role of vaccination. However, 34% of participants reported that the vaccine has not been fully tested and is not safe (Table 4). The VAX score was significantly higher in non-Qataris and those who received the influenza vaccine at least once (Table 5). Multiple linear regression showed that being non-Qatari and having received the influenza vaccine in the past were associated with higher vaccine hesitancy (Table 6).

**Table 1:** Demographic data and characteristics of participants (n = 325).

Demographic characteristics		Count	Column valid, %
Group	HMC healthcare worker	26	8.0
	General public	299	92.0
Nationality	Qatari	59	18.2
	Non-Qatari Arab	125	38.5
	Asian	62	19.1
	African	39	12.0
	European	26	8.0
	North American	14	4.3
	Central American	0	0.0
	South American	0	0.0
	Other	0	0.0
Educational level	High school	24	7.4
	Vocational training	12	3.7
	University	257	79.1
	Other	32	9.8
Occupation	Salaried	123	37.8
	Self-employed	46	14.2
	Unemployed	16	4.9
	Retired	140	43.1
Marital status	Single	22	6.8
	Married	303	93.2
Gender	Male	279	85.8
	Female	46	14.2

HMC = Hamad Medical Corporation.

**Table 2:** History of influenza vaccination, medical and psychiatric comorbidities, and personal or family COVID-19 infection.

Vaccination and medical/mental health statuses		Count	Column valid, %
Have you completed your childhood vaccinations?	Yes	255	84.7
	No	46	15.3
How often have you received the annual influenza vaccine in the past 3 y?	Annually	111	36.9
	Twice	44	14.6
	Once	38	12.6
	Never	108	35.9
Do you have any chronic medical illness?	Yes	195	64.8
	No	106	35.2
Diabetes	No	179	59.5
	Yes	122	40.5
Hypertension	No	156	51.8
	Yes	145	48.2
Dyslipidemia	No	255	84.7
	Yes	46	15.3
Asthma	No	287	95.3
	Yes	14	4.7
Ischemic heart disease	No	264	87.7
	Yes	37	12.3
Do you have any mental health illness?	Yes	5	1.7
	No	293	98.3
Are you on any regular medication?	Yes	245	87.2
	No	36	12.8
Have you or a family member had COVID-19?	I have had COVID-19	7	2.5
	A family member has had COVID-19	22	7.8
	Myself and at least 1 family member have had COVID-19	5	1.8
	No, neither myself or a family member have had COVID-19	247	87.9

COVID-19 = coronavirus disease 2019.

**Table 3:** Fears and worries associated with COVID-19.

COVID-19-associated fears and worries	Count	Column valid, %
Fear of becoming infected myself	128	39.4
Fear of a family member becoming infected	118	36.3
Financial worries	27	8.3
Job-related worries	21	6.5
No available vaccination yet	101	31.1
Somewhat worried	65	20.0
Not worried at all	68	20.9

COVID-19 = coronavirus disease 2019.

**Table 4:** Intention to accept COVID-19 vaccine and associated factors.

		Count	Column valid, %
Will you take the COVID-19 vaccine when it becomes available?	Definitely	143	50.9
	Probably	36	12.8
	Not sure	47	16.7
	Probably not	24	8.5
	Definitely not	31	11.0
What is the main reason for your willingness?	My understanding of the disease and vaccination	107	59.8
	Information from my doctor	30	16.8
	Information from social media	8	4.5
	Information from news	32	17.9
	Information from family/friends	2	1.1
When it becomes available, will you recommend the COVID-19 vaccine to elderly family members or family members with chronic conditions?	Definitely	142	50.9
	Probably	45	16.1
	Not sure	43	15.4
	Probably not	20	7.2
	Definitely not	29	10.4
If you have children, will you get your children vaccinated for COVID-19 when it becomes available?	Definitely	128	45.9
	Probably	43	15.4
	Not sure	53	19.0
	Probably not	20	7.2
	Definitely not	35	12.5
If you want to travel and the country of destination will waive the 2-wk quarantine period for those who got the COVID-19 vaccine, would you take the vaccine	I would definitely get the vaccine	127	51.4
	I would probably get the vaccine	61	24.7
	I would not take the vaccine and prefer to go through the quarantine requirements	59	23.9
COVID-19 is not a real disease	Strongly disagree	138	55.9
	2	19	7.7
	3	32	13.0
	4	21	8.5
	Strongly agree	37	15.0
COVID-19 is a new disease and vaccines against it have not been fully tested and will not be safe	Strongly disagree	58	23.5
	2	35	14.2
	3	41	16.6
	4	29	11.7
	Strongly agree	84	34.0

COVID-19 = coronavirus disease 2019.

**Table 5:** VAX mean scores in relation to demographic factors.

Demographic factors		Mean	Standard deviation	*P* value
Marital status	Single	31.54	10.24	.089
	Married	36.69	10.61	
Gender	Male	36.78	10.40	.203
	Female	34.33	11.87	
Higher education	No	35.20	9.96	.593
	Yes	36.53	10.71	
Qatari	No	37.35	10.39	.002
	Yes	31.73	10.76	
Have you completed your childhood vaccinations?	Yes	36.93	10.65	.065
	No	33.34	10.17	
Took influenza vaccine at least once	No	34.16	11.70	.012
	Yes	37.70	9.80	

VAX = Vaccine Attitudes Examination Scale.

**Table 6:** Stepwise multiple regression analysis of VAX scores in relation to demographic factors and personality dimensions.

Model		Unstandardized coefficients				95% Confidence interval for		
		β	Standard error	*t*	Significance	Lower bound	Upper bound	Partial correlations
1	(Constant)	34.759	1.182	29.417	.000	32.430	37.089	
	Took influenza vaccine at least once	3.871	1.498	2.584	.010	0.918	6.825	0.177
2	(Constant)	35.596	1.218	29.229	.000	33.195	37.997	
	Took influenza vaccine at least once	3.761	1.482	2.538	.012	0.840	6.682	0.174
	Qatari	–4.720	1.946	–2.425	.016	–8.558	–0.882	–0.167
			Excluded variables*			**Collinearity statistics**		
**Model**		**Beta In**	***t***	**Significance**	**Partial correlation**	**Tolerance**	**VIF**	**Minimum tolerance**
1	Gender	–0.056†	–0.820	.413	–0.057	0.999	1.001	0.999
	Higher education	0.036†	0.529	.598	0.037	0.988	1.012	0.988
	Marital status	0.078†	1.127	.261	0.078	0.984	1.016	0.984
	Qatari	–0.164†	–2.425	.016	–0.167	0.999	1.001	0.999
	Do you have any chronic medical illness?	0.081†	1.161	.247	0.081	0.951	1.051	0.951
	Do you have any mental health illness?	0.061†	0.896	.371	0.062	0.995	1.005	0.995
	Have you completed your childhood vaccinations?	–0.113†	–1.652	.100	–0.114	1.000	1.000	1.000
	Extraversion	–0.050†	–0.732	.465	–0.051	0.996	1.004	0.996
	Agreeableness	0.060†	0.877	.381	0.061	1.000	1.000	1.000
	Conscientiousness	0.034†	0.499	.618	0.035	0.998	1.002	0.998
	Emotional stability	0.026†	0.381	.703	0.027	0.992	1.008	0.992
	Openness to Experiences	0.051†	0.738	.461	0.051	1.000	1.000	1.000
2	Gender	–0.040‡	–0.590	.556	–0.041	0.989	1.011	0.989
	Higher education	0.018‡	0.260	.795	0.018	0.975	1.026	0.975
	Marital status	0.073‡	1.067	.287	0.074	0.983	1.017	0.983
	Do you have any chronic medical illness?	0.083‡	1.200	.232	0.084	0.951	1.051	0.951
	Do you have any chronic medical illness?	0.071‡	1.044	.298	0.073	0.992	1.008	0.992
	Have you completed your childhood vaccinations?	–0.095‡	–1.394	.165	-0.097	0.986	1.014	0.985
	Extraversion	–0.063‡	–0.927	.355	–0.065	0.990	1.010	0.990
	Agreeableness	0.034‡	0.498	.619	0.035	0.973	1.027	0.972
	Conscientiousness	0.016‡	0.236	.814	0.016	0.986	1.014	0.986
	Emotional stability	0.012‡	0.181	.857	0.013	0.984	1.016	0.984
	Openness to experiences	0.031‡	0.450	.653	0.031	0.984	1.016	0.984

Beta In = Beta coefficient, VAX = Vaccine Attitudes Examination Scale, VIF = variance inflation factor.

* Dependent variable: VAX.

† Predictors in the model: (constant), took influenza vaccine at least once.

‡ Predictors in the model: (constant), took influenza vaccine at least once, Qatari.

## 4. Discussion

In this study, we assessed attitudes toward the COVID-19 vaccine among the elderly population in Qatar. We found a 63.7% acceptance rate (50.9% would definitely and 12.8% would probably take the vaccine). The vaccine hesitancy rate (ie, respondents who were definitely or probably not going to take the vaccine) was 19.5%, similar to the 20.2% hesitancy rate in the general population.^[11]^ In comparison, 79.25% of individuals aged ≥45 years indicated that they would vaccinate against COVID-19 when it becomes available in Saudi Arabia, whereas 1.89% indicated that they would refuse vaccination, and 18.87% were unsure.^[16]^ Contoli et al^[19]^ found that out of 1876 respondents aged ≥65 years in Italy, 55% reported they would accept the vaccine, 16% would likely refuse, and 29% were hesitant. Furthermore, the national poll on healthy aging from the University of Michigan revealed that 58% of adults aged 50 to 80 years in the US would get the vaccine (33% very likely, 25% almost likely).^[20]^

About a third of participants were concerned about vaccine safety. Similarly, a qualitative study from southern Switzerland assessing the willingness of the elderly to vaccinate against COVID-19 found the majority in favor of inoculation, and participants against or unsure about the vaccine were mainly concerned about the novelty of the vaccine in addition to its safety and efficacy.^[21]^ It is important to note that our study was conducted before the availability of COVID-19 vaccines in Qatar. This can also explain why 31% were worried about vaccine unavailability. In December 2020, mRNA vaccines Moderna and Pfizer/BioNtech, both currently available in Qatar, received approval from The Food and Drug Administration through emergency authorization. Since then, studies showed high efficacy reaching 94% to 95% along with limited side effects and adverse reactions.^[22]^ Chemaitally et al^[23]^ showed that in Qatar, the Moderna vaccine was highly effective against different variants of SARS-CoV-2 and against COVID-19–related death and hospitalization, even after a single dose. Pfizer-BioNtech vaccine similarly yielded 97.4% effectiveness (95% confidence interval 92.2–99.5) against severe, critical, or fatal disease because of SARS-CoV-2 in Qatar.^[24]^ Reports on the high effectiveness and safety profiles of different COVID-19 vaccines should be harnessed in awareness campaigns to raise vaccine acceptance.

Among respondents, being non-Qatari predicted hesitancy. Gender, marital status, educational level, and socioeconomic status were not associated with significant differences in VAX scores. In comparison, older age groups and being married predicted vaccine acceptance in Saudi Arabia.^[16]^ The highest likelihood of vaccination against COVID-19 for individuals in Germany older than 75 years of age was associated with perceptions of vaccine efficacy because respondents with this belief were 4 times as likely to get vaccinated. Additional factors included the risk of becoming infected and the benefits of vaccination. The willingness to vaccinate was decreased by the vaccine’s adverse effects and general impediments to vaccination. Demographics associated with intention to receive vaccination included being single and having a history of chronic illness. Gender, educational level, and socioeconomic status were not associated with the intention to vaccinate.^[25]^ In the US, vaccine acceptance was more likely among those aged 65 to 80 years, males, being White compared to Hispanic and Black people, living with other people, having higher household income, and more education.^[20]^ Among older adults in Singapore, vaccine hesitators were more likely to be aged 71 to 75 years, of lower socioeconomic status, less likely to rely on official outlets for information on COVID-19, less trusting of all sources of information, having ≥1 chronic health conditions, and less socially integrated. Concerns mainly centered around vaccine safety and efficacy.^[26]^ Prioritizing nationals in vaccine campaigning and administration in Qatar might have contributed to the higher likelihood of nonnationals to be vaccine hesitators. In addition, information shared by the Ministry of Public Health through different media outlets regarding COVID-19 was mainly in the Arabic and English languages. As the population mainly comprised migrant workers from Asian countries such as India, Nepal, and Sri Lanka,^[27]^ the relative lack of publicly available information in their native languages might have contributed to the higher likelihood of vaccine hesitancy among non-Qataris.

Surprisingly, VAX scores were significantly higher among older individuals who have received the influenza vaccine at least once compared to those who never received it. In contrast, Martin and colleagues^14^ found significantly higher VAX scores among individuals who did not take the influenza vaccine in the previous year and those who intended not to take it in the same year the study was conducted. Similarly, vaccine hesitancy was associated with never having had the flu vaccine in a cross-sectional study involving a representative sample of Egyptian adults.^[28]^ Predictors of COVID-19 vaccine uncertainty included not having received the influenza vaccine the preceding year in addition to low income, poor adherence with government guidelines, female gender, and living with children in a large sample of United Kingdom adults.^[29]^ However, Iguacel and colleagues^[30]^ found no significant difference between vaccine hesitancy and prior influenza vaccination in a cross-sectional study in Spain involving adults aged ≥18 years. Bruin de Bruin et al^[31]^ showed that influenza vaccination behavior was associated with the perceived vaccine coverage and the beliefs and behaviors of one’s social circle (ie, friends and family). This might indicate that those who previously received the influenza vaccine were more attuned to attitudes of their social circle toward the COVID-19 vaccine and, accordingly, more hesitant during the study period when no one was yet vaccinated and concerns regarding vaccine efficacy and safety were prevalent.

The main factors driving the willingness to receive the COVID-19 vaccine among the elderly in Qatar were understanding the nature of disease and role of vaccination, followed by information provided by physicians. Fears mainly circled around vaccine safety. The paradigm shift should focus on highlighting accumulated findings on vaccine efficacy and safety, and educating physicians about their pivotal role in motivating wider vaccine acceptance. This is crucial because the Ministry of Public Health is currently campaigning the need for a booster or third dose of the COVID-19 vaccine.^[32]^ In addition, the emergence of SARS-CoV-2 B.1.1.529 variant, also known as Omicron,^[33]^ renders the eradication of COVID-19 unlikely in the near future, and efforts should be directed toward reducing its infectivity by advocating vaccines as a safe and effective means of protection in conjunction with other precautionary measures. Before vaccine availability, being non-Qatari and having received the influenza vaccine at least once predicted hesitancy. We recommend reassessing the psychosocial determinants of vaccine hesitancy among the elderly in Qatar following a year of awareness campaigning and vaccine administration to identify correlates hindering the acceptance of the third vaccine dose.

## 5. Strengths and limitations

Data were collected using an online questionnaire available only in Arabic and English. This might have excluded seniors who do not have access to the internet or have trouble using technology. Individuals who did not speak Arabic or English were additionally unable to participate. Our findings might also not reflect the current attitudes and willingness to accept the vaccine among the elderly because data were collected before vaccine availability. Social desirability bias could have also affected the results. Furthermore, although the number of participants was above the calculated minimum required sample size of 251, it might not be reflective of the older adult population in Qatar, with an estimated population size of about 30,000 individuals.^[17]^ The allocated time to submit responses was limited to 1 month (October 15 to November 15, 2020), and this could have contributed to the relatively low sample size in our study. Most respondents were predominantly non-Qatari males, which could have served as additional sources of potential bias in our results. However, this skewed distribution coincides with the demographics in the State of Qatar, in which nationals represent <15% of the total population,^[27]^ and individuals aged ≥65 years are predominantly males.^[17]^ Additionally, only Moderna and Pfizer-BioNtech vaccines were initially approved for inoculation against COVID-19 in the State of Qatar, and the generalizability of our findings to other World Health Organization–recommended vaccines might hence be limited. Nevertheless, our survey was widely distributed and publicized using different media platforms, and we used the VAX scale with validated Arabic translation, allowing us to compare our results to the existing literature.

## Author contributions

Mustafa Abdul Karim: conceptualization, preparation, writing-original draft. Shuja M. Reagu, Wesam S. Smidi, Nadeen Al-Baz: Data curation, writing-reviewing, and editing. Sami Ouanes: methodology, software, writing-reviewing, and editing. Shuja M. Reagu, Majid Alabdulla: conceptualization, methodology, supervision.
